# Reviews

**Published:** 1882-11

**Authors:** 


### Reviews.

**Nitro-Glycerine as a Remedy for Angina Pectoris.** By WM. MURRELL, M. D., M. R. C. P., Lecturer on Materia Medica and Therapeutics at the Westminster Hospital. Detroit, Mich.: George S. Davis, Medical Publisher. 1882.

Nitro-glycerine, or glonoine, which we think is the better name, has successfully passed through the stages of novelty and trial, and the more conservative practitioners are seeking for the most approved direction for its administration. This little work not only gives that, but the principal points are illustrated by reference to cases under the accomplished author's care. It is a timely and useful book.

---

**Social Equality: A Short Study in a Missing Science.** By WILLIAM HARRELL MALLOCK, Author of "Is life worth living?" New York: G. P. Putnam's Sons. 1882.

Although not in any sense a medical work, this is a book worth reading by physicians and other thinking men. The author takes the ground that any social changes that tend to abolish *inequalities* will tend also to destroy or diminish our civilization and happiness; that the idea of *equality*, as presented by the modern democrat, constitutes the most formidable danger that has ever threatened society; and that inequality, so far from being an evil of civilization, is the efficient cause of its development and present maintenance; that "labor is the source of all wealth and all culture." He most emphatically denies appealing to the actual facts of life to show that labor is *not* the ultimate cause of wealth, and that apart from other causes it would be utterly powerless to produce it, consequently that the distribution of wealth which the socialist programme demands is a scientific impossibility; and that no laws could accomplish it. Again we say the book is well worth the buying. Like all of Putnam's works, it is excellently printed with clear and easily-read type.

---

**The Physician Himself, and What he Should Add to his Scientific Acquirements.** By D. W. CATHELL, M. D., late Professor of Pathology, College of Physicians and Surgeons of Baltimore, Ex-President of the Medical and Surgical Society, active Member of the Medical and Chirurgical Faculty of Maryland, Honorary Member of the Lincoln Philosophical Society, etc., etc. Second edition, carefully revised. Baltimore, Md.: Published by Cushings & Bailey, 262 W. Baltimore St. By mail, $1.25.

We are glad to see that this work has met with sufficient approbation to call out, so soon, a second edition. The author has availed himself of the opportunity to enlarge, re-arrange and generally improve the work, and it is now more than ever worth the reading. The book is full of good common sense. An incongruity between the Doctor's advice and practice is, in one instance, apparent. On page 57 he very sensibly says: "When you publish anything, do not follow the custom of appending to your name a long tail consisting of all the titles and honors, whether strong or weak, that you can rake together, with half a dozen etcs. Such an enumeration is in bad taste and excites the ridicule of discerning people." On turning to the title page we find thereon the usual long list of empty titles, which, we entirely agree with the author, "is in bad taste," etc.

**Syphilis.** By V. CORNIL, Professor in the Faculty of Medicine of Paris, and Physician to the Lourcine Hospital. Translated, with notes and additions, by J. HENRY C. SIMES, M. D., Demonstrator of Pathological Histology in the University of Pennsylvania and Assistant Surgeon to the Episcopal Hospital, Philadelphia, and J. WILLIAM WHITE, M. D., Lecturer on Venereal Diseases and Demonstrator of Surgery in the University of Pennsylvania, and Surgeon to the Philadelphia Hospital. Fifty-eight illustrations. Philadelphia: Henry C. Lea's Son & Co. 1882.

This work is a valuable contribution to the literature of Syphilis, because it gives, to an extent unapproached by any other author, the minute anatomy and histological evolution of the various lesions of the disease, from the initial chancre to the gumma, including the mucous patch, the superficial and deep cutaneous syphilides, the osseous and visceral affections. The histological descriptions are largely original, and, as the author states, "I have spoken almost exclusively of what I have myself seen and investigated." The translators are to be praised for the manner in which they have performed their work, and their additions, which aggregate about one-third of the present volume, have added much to the practical value of the work by supplying additional clinical information on topics of importance. The illustrations are very good and sufficiently numerous.

---

**A Manual of Hypodermatic Medication: The Treatment of Diseases by the Hypodermatic Method.** By ROBERT S. BARTHOLOW, M. A., M. D., LL.D., Professor of Materia Medica and General Therapeutics in the Jefferson Medical College, of Philadelphia. Fourth edition, revised and enlarged. Philadelphia: J. B. Lippincott & Co. 1882.

The fourth edition of this valuable Manual is called for so soon after the issue of the previous edition that it is a more flattering comment upon its merits and value to the profession than can be given in any review we can write. Whatever Dr. Bartholow writes in the special department to which he has devoted his studies, is entitled to the highest consideration by the profession. In furnishing this Manual the same care and research and experience are observed as is found in all his productions. The Manual contains a history of subcutaneous medication, with a list of remedies, used hypodermatically. The author gives a review of the physiological action and of the therapeutical effect of each remedy. It will be seen that the work supplies a long-felt want, and the distinguished ability of the author stamps it with the authority which at once inspires confidence in its contents as a guide to this important method of medication.

---

**A Treatise on the Physiological and Therapeutical Action of the Sulphate of Quinine.** By Otis Frederick Manson, M. D., Professor of Physiology and Pathology in the Medical College of Virginia. Philadelphia: J. B. Lippincott & Co. 1882.

This small work of 164 pages contains the results of the author's investigations into the physiological and therapeutical action of this most important remedy of the materia medica. The field of labor in which Dr. Manson has acquired his experience, and from which he has drawn his conclusions is sufficiently fruitful in the diseases requiring cinchona and its alkaloids to entitle his manual to a careful perusal. Having given it such examination, we are prepared to recommend it to the profession who are searching for a wider scope in which to use the remedy to which this work is specially devoted.

---

**The International Encyclopædia of Surgery: a Systematic Treatise on the Theory and Practice of Surgery by Authors of Various Nations.** Edited by John Ashhurst, Jr., M. D., Professor of Clinical Surgery in the University of Pennsylvania. Illustrated with chromo-lithographs and wood-cuts. In six volumes. Volume ii. New York: William Wood & Co. 1882.

A recapitulation of the distinguished authors whom the editor has secured in the preparation of this valuable and exhaustive work, and their subjects, will afford the profession a clearer view of its value than we can impart in any other manner. *Contusions*—By Hunter McGuire, M. D., Medical College, Virginia. *Wounds*—By Thomas Bryant, F. R. C. S., Guy's Hospital, London. *The Antiseptic Method of Treating Wounds*—By W. Watson Cheyne, M. D., F. R. C. S., King's College Hospital, London. *Poisoned Wounds*—By John H. Packard, M. D., Episcopal Hospital and St. Joseph's Hospital, Philadelphia. *Sabre and Bayonet Wounds; Arrow Wounds*—By J. H. Bill, M. D., U. S. A. *Gun-Shot Wounds*—By P. S. Conner, M. D., Medical College of Ohio, Cincinnati. *The Effects of Heat*—By Thomas George Morton, M. D., Pennsylvania Hospital, Philadelphia. *The Effects of Cold*—By J. A. Grant, M. D., M. R. C. P., Lond., F. R. C. S., Edin., General Protestant Hospital, Ottawa. *Abscesses*—By Howard Marsh, F. R. C. S., St. Bartholomew's Hospital, London. *Ulcers*—By John T. Hodgen, M. D., LL. D., St. Louis Medical College. *Gangrene and Gangrenous Diseases*—By Edward M. Moore, M. D., University of Buffalo. *Venereal Diseases; Gonorrhœa*—By J. William White, M. D., University of Pennsylvania. *Venereal Diseases; The Simple Venereal Ulcer or Chancroid*—By F. R. Sturgis, M. D., University of the City of New York. *Venereal Diseases; Syphilis*—By Arthur Van Harlingen, M. D., Hospital of the University of Pennsylvania. *Venereal Diseases; Bubon D'Emblée; Venereal Warts or Vegetations; Pseudo-Venereal Affections; Venereal Diseases in the Lower Animals*—By H. R. Wharton, M. D., University of Pennsylvania. *Surgical Diseases of the Skin and its Appendages*—By James C. White, M. D., Professor of Dermatology in Harvard University. *Diseases of the Cellular Tissue*—By Joseph W. Howe, M. D., Bellevue Hospital Medical College. *Injuries and Diseases of the Bursæ*—By Charles B. Nancrede, M. D., Episcopal Hospital and W. Christopher's Hospital, Philadelphia. The work is printed upon the best quality of paper and contains 754 pages of matter. The list of authors and their subjects, which we have furnished, is a guaranty of its immense value as an addition to our surgical literature. The enterprising publishers in their efforts to bring out *the* surgical encyclopædia for the use of the profession deserve, as they are sure to receive, a liberal support and encouragement for their venture.

